# Risk factors for DSM 5 PTSD symptoms in Israeli civilians during the Gaza war

**DOI:** 10.1002/brb3.316

**Published:** 2015-02-12

**Authors:** Sharon Gil, Michael Weinberg, Keren Or-Chen, Hila Harel

**Affiliations:** Faculty of Social Welfare and Health Sciences, School of Social Work, University of HaifaMount Carmel, Haifa, 31905, Israel

**Keywords:** DSM 5, posttraumatic stress disorder (PTSD), risk factors, trauma

## Abstract

**Background:**

In light of the current modifications presented in the diagnostic criteria of posttraumatic stress disorder (PTSD) in the DSM 5, this study aimed at revalidating well-known PTSD risk factors, including gender, peritraumatic dissociation, social support, level of threat, and trait tendency for forgiveness.

**Method:**

Five hundred and one Israeli civilians were assessed during real-time exposure to missile and rocket fire at the eruption of the Gaza war. Assessments took place approximately one to 2 weeks after the beginning of this military operation, relying on web administration of the study, which allowed simultaneous data collection from respondents in the three regions in Israel that were under attack.

**Results:**

A structural equation model design revealed that higher levels of forgiveness toward situations were associated with fewer PTSD symptoms, whereas peritraumatic dissociation and high levels of objective and subjective threat were positively associated with PTSD symptoms. Additionally, females were at higher risk for PTSD symptoms than males.

**Conclusions:**

The findings of this study provide further evidence for the importance of directing preventive attention to those vulnerable to the development of elevated levels of PTSD symptoms. Theoretical and clinical implications of the findings are discussed.

## Introduction

Foremost among the significant modifications of diagnostic criteria for posttraumatic stress disorder (PTSD) in the 5th edition of the Diagnostic and Statistical Manual of Mental Disorders (American Psychiatric Association 2013) was the relocation of PTSD into a new class of trauma and stressor-related disorders. Criterion A in the DSM-5 specifies events with traumatic potential (threat of, or actual exposure to death, physical assault, or sexual violation) while omitting the previous criteria of fear, helplessness, or horror in reaction to the traumatic exposure (American Psychiatric Association 1994). Additionally, a new symptom cluster was added: Criterion D, namely negative alterations in cognition and mood, and changes in the avoidance and hyper-arousal clusters (Friedman et al. 2011a; American Psychiatric Association 2013).

Posttraumatic stress disorder follows and is a result of exposure to a traumatic event; however, the exposure by itself is not a sufficient precondition for the onset of PTSD (Friedman et al. 2011b), leading investigators to examine risk factors for the development of PTSD following such exposure. Studies in the field (e.g., Ozer et al. 2003; Johnson and Thompson 2008; Johnson et al. 2009) have identified several significant variables as risk factors, mainly a history of traumatic exposure, age, and gender (with female at higher risk for PTSD), as well as genetic factors (e.g., Ballenger et al. 2000; Norris et al. 2002; Kessler et al. 2005).

Another pretraumatic factor that has attracted growing research attention in recent decades is the individual's personality traits (e.g., Brewin et al. 2000; Jaksic et al. 2012; Gil 2005(. Trauma victims seeking to relieve ongoing stress posed by the exposure make efforts to cope with the stress (Lazarus 1999). While Lazarus's transactional model mainly emphasizes situational factors, deepening the study of the coping process has demonstrated that the effort individuals make may depend not only on the type of situational demand but also on their personality traits – referred to as internal resources or personality trait (Lazarus 1999; Connor-Smith and Flachsbart 2007; Carver and Connor-Smith 2010). Within this context, relations between personality and coping were generally stronger in samples facing a high degree of stress (e.g., cancer, acute or chronic trauma) than in samples with little stress (Connor-Smith and Flachsbart 2007).

An internal resource that has attracted growing interest among researchers in the field of dealing with stressful situations is forgiveness (Enright 1991; Brown 2003; Witvliet et al. 2004; Snyder and Heinze 2005; Weinberg et al. 2014). The notion of forgiveness encompasses cognitive, emotional, and behavioral acts in response to a transgression (Fincham and Kashdan 2004), transforming negative emotions into neutral or positive ones (Thompson et al. 2005). Forgiveness, however, is not equated with forgetting, pardoning, condoning, excusing, denying the offense, or conflict resolution (Worthington et al. 2007).

Forgiveness is divided as trait forgiveness (a personality tendency to remain stable in the face of stress and trauma) and state forgiveness (a tendency to react to stress and trauma) (Maltby et al. 2004; Bono et al. 2008). Research has demonstrated that forgiving individuals are more optimistic, outgoing, and confident. Individuals who are not forgiving demonstrate anxiety, worry, and moody personality traits, and are not likely to engage in or acknowledge stressful events (Hall and Fincham 2005). More recently, forgiveness has been associated with improvement in mental health, physical health, self-esteem, well-being, and life satisfaction (Strelan 2007). In particular, research in this area has demonstrated the positive effect of tendency to forgive on well-being (Heinze and Snyder 2001).

Additionally, multiple dimensions of tendency to forgive have been addressed, such as self-forgiveness, whereby the individual shifts from being negatively motivated to positively motivated toward him/herself (Kaminer et al. 2001), and situational forgiveness, which refers to the source of a perceived transgression that might not be readily attributed to another person, or oneself, but rather to “an unjust world,” “life,” or “fate” (Friedberg et al. 2005). In this context, forgiveness of self and situations appears to be more strongly related to aspects of psychological well-being than to forgiveness of others (Heinze and Snyder 2001). Among people who have experienced traumas, forgiveness of self and situations (but not forgiveness of others) were significantly negatively correlated with symptoms of PTSD (Hamama-Raz et al. 2008).

Recent research literature examining the relationship between forgiveness and PTSD symptoms among trauma victims has revealed an association between higher levels of forgiveness and lower levels of PTSD symptoms (Heinze and Snyder 2001). A study of individuals in South Africa who had suffered violations of their human rights (e.g., murder of a family member, abduction, torture) demonstrated that victims who were less inclined to forgive showed higher levels of depression, PTSD symptoms, and other anxiety disorders (Kaminer et al. 2001). In the context of terror attacks, Friedberg et al. (2005) found that lower levels of tendency to forgive predicted perceived stress, but not trauma, among New York City residents. Hamama-Raz et al. (2008) showed that among high school students in Israel, a low tendency to forgive was associated with high levels of PTSD symptoms.

However, the current modifications in the DSM 5 call for reevaluating the association between levels of DSM 5 PTSD symptoms with previously well-established PTSD risk factors. This study, therefore, examined gender, peritraumatic dissociation, social support, levels of threat, and trait tendency for forgiveness as PTSD risk factors for DSM 5 symptoms. Five hundred and one Israeli civilians were assessed during the Gaza war starting 2 weeks after the beginning of the operation, during which over 4500 rockets were fired from the Gaza Strip toward large areas of Israel, and 67 Israeli soldiers, 5 Israeli civilians, and over 2200 Hamas operatives and Gaza civilians in the Gaza Strip were killed. Massive destruction of property and a continuous feeling of threat and hazard on both sides of the border existed throughout the period.

The following hypotheses were posited:

1. A positive association will be found between levels of objective and subjective threat and levels of PTSD symptoms.
2. A negative association will be found between peritraumatic dissociation and levels of PTSD symptoms.
3. A negative association will be found between forgiveness to situation and levels of PTSD symptoms, whereas no association will be observed between forgiveness to self and others and levels of PTSD symptoms.
4. A negative association will be found between social support and levels of PTSD.

## Method

### Participants and procedure

The data in this study is based on real-time responses of Israeli civilians during an ongoing period of threat of missile and rocket fire. The sample consisted of 501 residents living within the rocket range, divided into three groups: 13.4% (*n *=* *67) living 7–40 km from the border, 32.93% (*n *=* *165) living 40–80 km from the border, and 53.7% (*n *=* *269) living more than 80 km from the border. All three areas were under constant attack. The time available to reach a shelter between the air-raid siren warning of incoming fire, and the impact of the missiles, varied depending on distance from the Gaza Strip border. The amount of warning ranged from 15 s or less in the border areas to 90 s in areas that were over 80 km from the border. The definition of threat in the study was based on the Israeli HFC (Home Front Command) division, which calculated the extent of time an individual had in order to get to a shelter from the moment the red siren started until the rocket(s) fell. This time span signified the degree of threat to each individual – the less time one had, the higher his/her chances to be physically or mentally injured.

As the study aimed to investigate respondents’ reports in real time under ongoing life-threatening conditions, the authors decided to rely on a web administration of the study, which allowed simultaneous data collection from respondents in the three regions in Israel that were under attack. Consequently, a snowball sampling technique was chosen utilizing Internet-based social media outlets to invite individuals in the specified areas of Israel to participate in the study, and suggesting that they invite friends and family members to take part in the study as well. Data collection started approximately 2 weeks after the beginning of the operation and ended 4 weeks later following the first 72-h ceasefire declaration by the U.N.

The study was approved by the Haifa University Ethics Committee, ensuring privacy and confidentiality. All participants provided electronic informed consent before participating in the study.

Of the participants, 97 (19.5%) were male and 404 (80.5%) were female; mean age 37.32 (SD = 10.74); mean years of education 17.10 (SD = 5.15). The relatively low rates of male respondents may be attributed to their military recruitment.

## Measures

### Demographic questionnaire

This covered variables such as gender, age, education, family statues, religion, and occupation.

### Objective and subjective levels of threat

The objective threat was defined by the time available to take shelter. Three levels of threat were defined: (1) High Exposure Severity: 7–40 km from the border; (2) Moderate Exposure Severity: 40–80 km from the border; and (3) Low Exposure Severity: over 80 km from the border.

The subjective threat was examined by a single question: “In your estimation, is there a real threat that missiles will harm your living space The answer was rated on a 4-point Likert scale ranging from *1 = not at all* to *4 = great risk*.

### Trait Tendency to forgive

Tendency to forgive was examined using the Heartland Forgiveness Scale (HFS; Fincham and Kashdan 2004). This questionnaire includes 18 items rated on a seven-point Likert scale ranging from 1 = *not appropriate* to 7 = *very appropriate*, including three subscales that examine trait forgiveness of self, others, and situations. Thompson's study (Fincham and Kashdan 2004) showed internal consistency for the subscales, ranging from 0.72 to 0.87. Tendencies to forgive self, others, and situations were measured by the average overall score of the subscales. Higher scores represented a stronger tendency to forgive. The Cronbach's alpha internal consistency for subscales measuring forgiveness of self, others, and situation were 0.68, 0.81, and 82, respectively. The Cronbach alpha internal consistency for the general scale was 0.86.

### Peritraumatic dissociation

The Peritraumatic Dissociative Experiences Questionnaire (PTDQ; Hamama-Raz et al. 2008) was used to assess the extent to which dissociation had been experienced during the air-raid sirens warning of incoming fire and missile attacks. The questionnaire includes eight items rated on a five-point Likert scale ranging from 1 = *not at all true* to 5 = *extremely true*. The Cronbach's alpha internal consistency for the scale was 0.88.

### Social support

Social support was evaluated using the Multidimensional Scale of Perceived Social Support (MSPSS; Marmar et al. 1994). The questionnaire includes 12 items and relates to three sources of social support: family, friends, and significant other. The items are rated on a seven-point Likert scale ranging from 1 = *major disagreement* to 7 = *major agreement*. The Cronbach alpha internal consistency for the subscales measuring support from family, friends and significant other were 0.92, 0.92, and 0.87 respectively. The Cronbach alpha internal consistency for the general scale was 0.93.

### DSM 5 PSTD Symptom Levels Scale (PSLS)

Posttraumatic stress disorder symptoms were evaluated by a questionnaire compiled by the authors, corresponding fully with the DSM 5 (American Psychiatric Association 2013) criteria for PTSD (PSTD Symptom Levels; PSLS, Appendix 1 1994). The questionnaire adheres to the construct and expert validity of the DSM 5 diagnosis of PTSD. It is a 20-item self-report questionnaire aimed at assessing levels of PTSD symptoms over the preceding 2 weeks. Each item corresponds to one of the 20 DSM 5 diagnostic criteria for PTSD. The severity of each item is rated on a four-point Likert scale ranging from 0 = *not at all* to 3 = *severely*. The total severity score is calculated as the mean of the respondent's ratings of the 20 items. The scale is divided into four clusters: intrusion (items 1–5), avoidance (items 6–7), negative alterations (items 8–14), and alterations in arousal (items 15–20). The scale showed high Cronbach alpha internal consistency for both the general scale and for its subscales.

Validity and reliability of the DSM 5 PSTD Symptom Levels Scale (PSLS) Construct validity: The PSLS was developed in adherence to the diagnostic criteria presented in the DSM-5. Each question corresponds to the 20 items developed by experts; thus, construct validity is maintained.

1. Content validity: Twenty psychiatrists from three mental health hospitals in Israel, were asked to rate the extent to which the questionnaire matched the DSM 5 PTSD diagnostic criteria on a Likert scale ranging from 1 = *very low match* to 5 = *very high match*. The mean score of their rating was 4.7 (SD = 0.8), indicating a high content validity.
2. Internal validity: Pearson product correlation coefficients, conducted to identify correlations between the total PSLS score and its 20 items, ranged from (*r* = 0.91; *P* < 0.001) for item 1 to (*r* = 0.63; *P* < 0.001) for item 10. In addition, Pearson product correlation coefficients showed intrusion and strong correlation between the four clusters and the PSLS total score (*r* = 0.86; *P* < 0.001), avoidance (*r* = 0.73; *P* < 0.001), negative alterations (*r* = 0.87; *P* < 0.001), and arousal (*r* = 0.91; *P* < 0.001).
3. External validation: In order to examine external validity, the correlation between the PSLS and a well-established well-being questionnaire (Zimet et al. 1988; an opposite external indicator) were tested. The high negative association between these two tools indicates a high external validity of the PSLS.
4. Criterion validity: Twenty of the respondents were recruited randomly immediately after completing the Internet-based questionnaire, and agreed to be examined by a psychiatrist. Only three received a formal diagnosis of ASD, but their scores on the PSLS were indeed relatively high (M = 0.53, SD = 0.54) compared to the total mean score of the entire sample (M = 1.1, SD = 0.3). Clearly, no formal statistical analysis is suitable.
5. Reliability: The scale showed high Cronbach alpha internal consistency for both the general scale and for its subscales. The Cronbach alpha internal consistency for the intrusion subscale was 0.87, for the avoidance subscale 0.84, for the negative alterations subscale 0.86, and for the alterations in arousal subscale 0.85. The Cronbach alpha internal consistency for the general scale was 0.94. In addition, Pearson product correlation coefficients, conducted to identify correlations between the total PSL score and its subscales, showed intrusion (*r* = 0.86; *P *<* *0.001), avoidance (*r* = 0.73; *P *<* *0.001), negative alterations (*r* = 0.87; *P *<* *0.001), and arousal (*r* = 0.91; *P *<* *0.001).

## Results

Data analyses were performed in two stages, corresponding to the aim of the study. In the first stage, SPSS 21 was used to examine the relationship between levels of PTSD symptoms and risk factors for PTSD. Pearson product correlation coefficients revealed a negative relationship between PTSD symptoms and forgiveness to the situation (*r* = −0.41; *P *<* *0.001), forgiveness to self (*r* = −0.24; *P *<* *0.001), forgiveness to others (*r* = −0.15; *P* < 0.001), level of objective threat (*r* = −0.30; *P *<* *0.001), and social support (*r* = −0.16; *P *<* *0.001). Conversely, a positive relationship was found between PTSD symptoms and peritraumatic dissociation (*r* = 0.58; *P *<* *0.001), objective levels of threat (*r* = 0.49; *P* < 0.001), subjective levels of threat (*r* = 0.34; *P *<* *0.001), and gender (*r* = 0.18; *P *<* *0.001, with females being at a higher risk).

**Figure 1:**
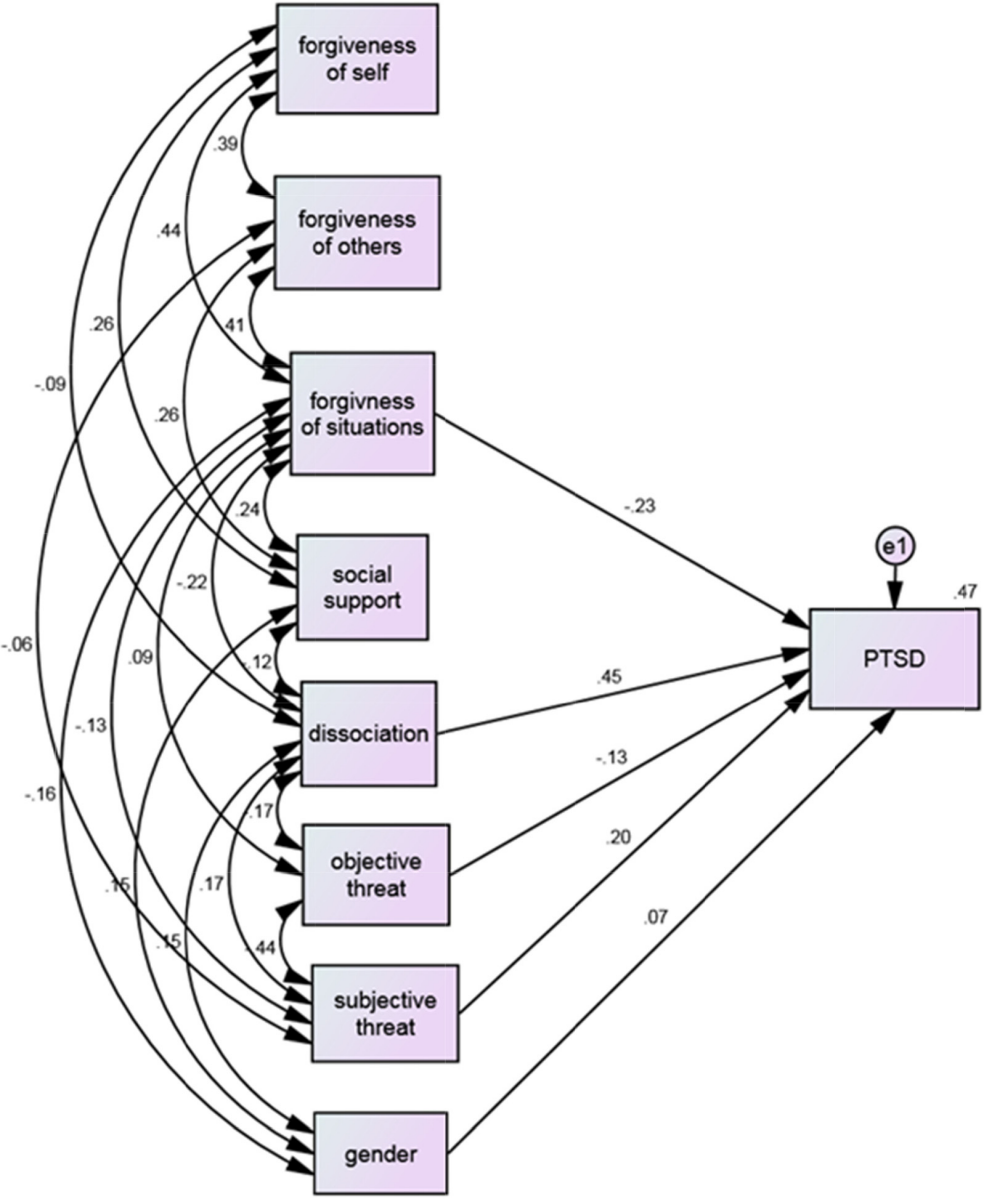
Structural equation model (SEM) for PTSD risk factors, e1 = dependent variable. *Note*: With regard to the significance of the associations between the risk factors and PTSD symptoms, for all of the associations *P *<* *0.001 except for gender (*P *=* *0.03). With regard to the significance of correlations between the risk factors, the significance for all of the correlations were *P *<* *0.001, except for: dissociation and social support (*P = *0.004), dissociation and forgiveness of self (*P *=* *0.02), and forgiveness of situations and objective threat (*P *=* *0.02).

The second stage examined the associations posited in the research hypotheses when applied to a nested structural equation model (SEM) design (Fig.1).

Analyses were conducted with AMOS (version 18; Cummins and Lau 2005) using the maximum-likelihood method. Nonsignificant paths were deleted. In addition to the overall test of exact fit, the following fit indices were used to evaluate the proposed models: (a) the *χ*^2^/df ratio, (b) the root mean square error of approximation (RMSEA), (c) the comparative fit index (CFI), and (d) the nonnormed fit index (NNFI). A model in which *χ*^2^/df was ≤2, CFI and NNFI were >0.90, and the RMSEA index was between 0.00 and 0.09 (Arbuckle 2009) was deemed acceptable. These moderately stringent acceptance criteria clearly reject inadequate or poorly specified models but they accept models for consideration that meet real-world criteria for reasonable fit and representation of the data (Hu and Bentler 1999). The present model fit the observed data very well [*χ*^2^ (df* = *14) = 19.384, *P *=* *0.151, *χ*^2^/df* =* 1.38] with a good fit measure (NNFI = 0.978, CFI = 0.994, RMSEA = 0.028).

As indicated in Figure1, a higher tendency to forgive situations is associated with fewer PTSD symptoms. In addition, higher peritraumatic dissociation is associated with higher PTSD symptoms. While taking into account objective threat and PTSD symptoms, the model reveals that the further the individuals were from the Gaza Strip border, the fewer PTSD symptoms they demonstrated. A positive association was found between subjective threat and PTSD symptoms for all associations (*P *<* *0.001). Females are associated with higher PTSD symptoms than males (*P *=* *0.03). Notably, social support and tendency to forgive self and others failed to demonstrate a significant association with PTSD symptoms. The significance for all the correlations between the risk factors presented (Fig.1) was *P *<* *0.001, except for the correlations between dissociation and social support (*P = *0.004), dissociation and forgiveness of self (*P *=* *0.02), and forgiveness of situations and objective threat (*P *=* *0.2). The model explained 47% of the variance in PTSD symptoms.

## Discussion

The 5th edition of the Diagnostic and Statistical Manual of Mental Disorders (DSM 5; American Psychiatric Association 1994) presented several significant modifications (American Psychiatric Association 1994, 2013; Friedman et al. 2011a,b) of the diagnostic criteria for PTSD. As a result, previous well-known PTSD risk factors require reevaluation.

This study aimed at reevaluating the association between levels of DSM 5 PTSD symptoms with several of these variables, including, gender, peritraumatic dissociation, social support, levels of objective and subjective threat, and trait tendency for forgiveness – variables which have been associated with PTSD symptoms in previous studies prior to modifications in the DSM 5 for PTSD (Ballenger et al. 2000; Brewin et al. 2000; Norris et al. 2002; Ozer et al. 2003; Gil 2005; Kessler et al. 2005; Johnson et al. 2009; Jaksic et al. 2012). Specifically, 501 Israeli civilians living in missile attack areas were assessed during the ongoing Gaza war. Recruitment was conducted by Internet-based social media outlets, starting 2 weeks after the beginning of the operation, over approximately a 1-month period.

As hypothesized, this study validates that DSM 5 PTSD symptoms increase in reaction to both objective and subjective threat, reinforcing the current notion that trauma is not solely an objective occurrence (Lazarus 1999; Brewin et al. 2000), but rather is a combination of exposure to an actual event and a subjective perception of that event as traumatic.

The finding also supports the linkage between personality traits and PTSD (Lazarus 1999; Brewin et al. 2000; Gil 2005; Connor-Smith and Flachsbart 2007; Carver and Connor-Smith 2010; Jaksic et al. 2012). Such traits encompass characteristic patterns of thought, feelings, and actions consistent across developmental periods and contexts. The underlying assumption is that individual differences in personality structure dictate vulnerability/resilience to the development and formation of PTSD symptoms among traumatized individuals.

Specifically, our findings confirm the study hypothesis that a high tendency to forgive is associated with a reduction in PTSD symptoms, as demonstrated in previous findings (Heinze and Snyder 2001; Kaminer et al. 2001; Witvliet et al. 2004; Friedberg et al. 2005; Snyder and Heinze 2005; Hamama-Raz et al. 2008; Weinberg et al. 2014). Specifically, the findings reveal that under life-threatening conditions such as war, situational forgiveness had the strongest impact on PTSD symptom reduction, whereas forgiveness to self or others showed no association with the risk for PTSD symptoms. Similar to other personality traits, trait forgiveness is a stable individual attribute (Friedberg et al. 2005; Jaksic et al. 2012) that can serve as an internal resource when dealing with stressful situations. Hence, trait forgiveness in this study may be assumed to predict a reduction in PTSD symptoms levels even in the face of prolonged war.

Moreover, while a condition of war such as described above is beyond the influence of the civilian or his/her relationship with others, it appears reasonable that a primary mode of adjustment draws on a trait of forgiveness to situations, thus improving one's sense of mastery (Lazarus 1999). Previous research has demonstrated that a personal tendency to forgive situations (Weinberg et al. 2014) and a heightened level of mastery (Carver and Connor-Smith 2010) protects victims of terror attacks against PTSD symptoms. Conceivably, in the context of war, when civilians are under continuous life-threatening rocket and missile fire, situational aspects have greater impact than intra- and interpersonal dimensions. Given that the individuals have no control over the missile strikes, and taking into account that there is no connection with those firing at them, civilians perceive the incidents as random, fate, or chance. Notably, however, as the tendency to forgive situations is a personality trait, people who are less inclined to forgive situations are at a higher risk for PTSD and require special preventive attention.

Surprisingly, in contrast to the research hypothesis, peritraumatic dissociation was found to increase, rather than reduce, the risk for PTSD symptoms. This discrepancy might be explained by the well-established notion that peritraumatic dissociation is often a transient reaction during exposure to a traumatic event. Although its immediate effects are assumed to be adaptive, pathological outcomes may appear in the long term, particularly when the reaction is prolonged (Ballenger et al. 2000; Brewin et al. 2000; Norris et al. 2002; Ozer et al. 2003; Kessler et al. 2005). Since the Gaza war was a continuous uncertain condition, posing a prolonged threat to civilians, their dissociative reaction might be prolonged as well, thus increasing the risk for PTSD. Indeed, dissociation in this study was found to decrease levels of forgiveness to situations and increase objective and subjective threat.

Likewise, social support, which is generally considered a protective factor against PTSD (Lazarus 1999; Carver and Connor-Smith 2010), may be less effective in a prolonged mass threat, with its effect reduced over time. Moreover, and with all due caution, the issue of the justification of the operation within the Israeli population and around the world may have also contributed to a lack of association between social support and reduced PTSD symptoms. These speculations clearly need further examination.

Together, the findings have clinical and practical implications which constitute a tangible contribution. The findings of the study revalidate the consistent role of several well-known PTSD risk and protective factors regardless of the modifications made in the DSM 5 diagnostic criteria, namely: gender (with female at higher risk for PTSD symptoms), objective and subjective threat as positive risk factors, personality trait of forgiveness to situations as a protective factor, and prolonged dissociation as a negative risk factor.

Clearly, these conclusions must be treated with caution, considering several of this study's limitations. First, generalization is restricted to Israeli civilians experiencing an ongoing threat, damage, and loss of life by missile attacks and nearby continuous combat, resulting in property damage and killing of civilians in the Gaza Strip as well as Israeli soldiers. Second, Internet-based recruitment is inherently biased in favor of Internet responders. Third, the relatively low rates of male respondents may be attributed to their military recruitment. Still, 97 (19.5%) male respondents is not a negligible amount. Moreover, our findings that females are at a higher risk for PTSD symptoms is consistent with the findings of most studies in the field, thus it does not appear that the low rates of male respondents affect our findings. Lastly, PTSD symptoms in the study were assessed by an original instrument corresponding to a revised version of the diagnostic criteria of the PTSD (American Psychiatric Association 2013). Although its validity and reliability in this study are not in doubt, further examinations are necessary. In light of the circumstance of recruiting individuals under fire, the limitations associated with this sampling procedure seem to constitute an acceptable trade-off.

Despite these limitations, the findings of the study are of significance in applying the revised version of the PTSD DSM 5 diagnostic criteria in a unique real-time combat situation, thereby directing preventive attention to individuals vulnerable to the development of elevated levels of PTSD symptoms in the short and long run.

## Conflict of Interest

None declared.

## Appendix 1: DSM 5 PSTD Symptom Levels Scale (PSLS)

**Instructions:** Below is a list of symptoms that people sometimes have in response to stressful or traumatic life experiences. With regard to the current Gaza war, please circle one of the numbers in the scale to indicate the extent to which you have been troubled by these symptoms during the past 1 week.

**Table 1:**

	Not at all	Slightly	Moderately	Severely
1. Repeated, involuntary, and intrusive disturbing memories of the stressful experience	0	1	2	3
2. Repeated, disturbing dreams of the stressful experience	0	1	2	3
3. Feeling or acting as if the stressful experience is happening again (as if you were reliving it)	0	1	2	3
4. Intense prolonged psychological distress (e.g., emotional distress, feeling sad, upset) when something reminded you of the stressful experience	0	1	2	3
5. Marked physical reactions (e.g., pounding heartbeat, lack of air while breathing, sweating) when something reminded you of the stressful experience	0	1	2	3
6. Avoiding thinking about or talking about the stressful experience or avoiding having feelings related to it	0	1	2	3
7. Avoiding activities or situations (e.g., people, places, conversations, activities, objects) because they reminded you of the stressful experience	0	1	2	3
8. Trouble remembering important parts of the stressful experience	0	1	2	3
9. Negative beliefs or expectations about yourself or the world (e.g., I am bad, no one can be trusted, the world is a completely dangerous, my nervous system is permanently ruined)	0	1	2	3
10. Thoughts that the circumstances that led to the stressful situation was your fault	0	1	2	3
11. Persistent negative emotional state (e.g., fear, horror, guilt or shame)	0	1	2	3
12. Loss of interest in activities that you used to enjoy	0	1	2	3
13. Feeling distant or cut off from other people	0	1	2	3
14. Feeling emotionally numb or being unable to have positive emotions such as happiness, satisfaction, or love	0	1	2	3
15. Irritable behavior and angry outbursts (with little or no provocation), such as verbal or physical aggression toward people or objects	0	1	2	3
16. Reckless or self-destructive behavior	0	1	2	3
17. Being “super alert,” watchful, or on guard	0	1	2	3
18. Feeling jumpy or easily startled	0	1	2	3
19. Having difficulty concentrating	0	1	2	3
20. Trouble falling or staying asleep	0	1	2	3
